# Rapid Screening and Quantitative Determination of Illegal Phosphodiesterase Type 5 Inhibitors (PDE-5i) in Herbal Dietary Supplements

**DOI:** 10.1155/2021/5579500

**Published:** 2021-05-05

**Authors:** Thi Oanh Nguyen, Cao-Son Tran, Thi Thu Hang Do, Thi Minh Hoa Nguyen, Quang-Dong Bui, Cao-Tien Bui, Hong-Ngoc Nguyen, Thu-Hien Dang, Viet-Chien Dinh, Thi Anh Huong Nguyen, Thi Hong Hao Le

**Affiliations:** ^1^Vietnam Food Administration, 135 Nui Truc, Ba Dinh, Hanoi 10000, Vietnam; ^2^University of Science, Vietnam National University, 334 Nguyen Trai, Thanh Xuan, Hanoi 10000, Vietnam; ^3^National Institute for Food Control, 65 Pham Than Duat, Cau Giay, Hanoi 10000, Vietnam

## Abstract

Phosphodiesterase type 5 inhibitors (PDE-5i) are the first-line medication for oral erectile dysfunction, which are used according to the prescription of doctors. However, these substances have been found illegally in supplementary foods. The quality and safety of dietary supplements for enhancing male sexual performance have been questioned, raising the need for continual development of analytical methods. Liquid chromatography coupled with high-resolution mass spectrometry has become one of the most effective methods to identify and measure PDE-5i concentration. In this research, we focused on (i) developing and validating an effective screening and quantitation method for more than 53 PDE-5i in ingredients and supplementary products using LC-Q-Exactive after a simple sample extraction and (ii) assessing PDE-5i content in natural-based supplementary products available in Vietnam market. The extraction method used a small amount of organic solvent, which makes it more environmentally friendly (greener). The developed method has a limit of detection of 0.4 mg/kg, a limit of quantitation of 1.2 mg/kg, recoveries from 80 to 110%, and repeatability lower than 15%. Ninety-two herbal supplementary foods and ingredients used for enhancement of male sexual performance available in Vietnamese markets were collected. Fourteen PDE-5i including conventional and novel analogous were detected and measured in eighteen food supplements and two formulation ingredient samples.

## 1. Introduction

Phosphodiesterase type 5 (PDE-5) is an enzyme responsible for the breakdown of cyclic guanosine monophosphate (cGMP) in the corporal smooth muscle [1]. Thus, PDE-5i are considered the first-line medication for oral erectile dysfunction (ED) therapies [2]. After the approval of sildenafil, several PDE-5i have been approved and demonstrated well-established efficacy in patients with ED such as tadalafil, vardenafil, avanafil, mirodenafil, undenafil, and lodenafil. Additionally, PDE-5i proved their great potential in the treatment of neuroinflammation, neurodegeneration, cognition (Alzheimer's disease), cancer therapeutics, diabetic peripheral neuropathy, renoprotection, etc. [3]. Although the safety of PDE-5i was proven, the use of PDE-5i has some adverse effects such as ataxia caused by acetildenafil and its analogs and symptoms of giddiness, headache, shortness of breath, and backache [4]. PDE-5i have an interaction effect with other medicines such as nitrates. From 2007, the US FDA announced that a warning of the potential risk of visual and auditory impairment related to nonarteritic anterior ischemic optic neuropathy and sudden sensorineural hearing loss would be added to drug labels of PDE-5i [3]. Seriously, the first known fatal case related to desmethyl carbodenafil, an unapproved PDE-5i, on a 34-year old male was reported in 2017 [5]. Therefore, the use of PDE-5i in therapy should strictly obey the advice of pharmacists.

PDE-5i, namely sildenafil, tadalafil, vardenafil, and their analogs, have been added in supplementary foods, which were supposed to be made of natural ingredients. The presence of PDE-5i in these supplement foods without labeling is consumer deception. In 2013, J.H. Lee et al. reported more than 46 PDE-5i analogs in various forms of health food products in the online and offline market of Korea [6]. A case study in the Czech market revealed that 10 over 64 natural herbal-based supplements for ED treatment contain both registered or unregistered synthetic PDE-5i [7]. In the summary of the Min-Yong Low research group, Asia reported the highest number of PDE-5i as adulterants in dietary supplements and was followed by Europe and North America [4]. Research of the Malaysian market showed 82% tested unregistered products and 14% of the registered products were adulterated with PDE-5i or their analogs [8]. The regulation of PDE-5i has been complicated because of the increasing number of novel synthetic PDE-5i analogs. Thus, the current situation raises the need for continual development of analytical methods to quickly detect PDE-5i analogs in these products.

The rapid and accurate identification and measurement of popular and unknown PDE-5i have been improved by numerous analytical techniques including high-performance liquid chromatography (HPLC) [9, 10], gas chromatography-mass spectrometry (GC-MS), nuclear magnetic resonance (NMR) spectroscopy [11], vibrational spectroscopy, liquid chromatography-Fourier transform ion cyclotron resonance mass spectrometry (LC-FT-ICR-MS), etc. [12]. The most effective approach for the identification of PDE-5i in supplements is HLPC-MS [6, 13, 14], although some publications had been done by HPLC with a UV detector [9] or photodiode array detector [14]. In 2015, the AOAC International published an official method for screening and identification of PDE-5i in dietary ingredients and supplements [15]. The samples were simply extracted with a mixture of solvent (methanol, acetonitrile, water), then diluted, filtered, and analyzed by LC quadrupole-orbital ion trap MS. Identification of targeted and nontargeted analytes was conducted based on retention time, accurate mass, and isotopic pattern of precursors ions and product ions using an in-house database. Recently, Hong et al. have reported a screening and classification method of PDE-5i by GC-MS [16]. Specific common ions according to structural after the trimethylsilyl derivatization characteristics of four PDE-5i classes were found.

The development of high-resolution mass spectrometry (HRMS) techniques has made screening applications more selective than conventional MS techniques. Currently, time-of-flight mass spectrometry (TOF) or Orbitrap mass spectrometry techniques can achieve high mass accuracy (below 5 ppm). In particular, Orbitrap mass spectrometry can perform high-resolution MS/MS allows both the screening of unknown compounds and the quantification of target substances. Therefore, liquid chromatography coupled with HRMS has become one of the most effective methods to identify and measure PDE-5i concentration. Our goals are (i) developing and validating a screening and quantitation method for PDE-5i in supplementary products and (ii) assessing PDE-5i content in natural-based supplementary products and ingredients available in the Vietnam market.

## 2. Materials and Methods

### 2.1. Chemicals and Reagents

Fifty-three PDE-5i standards were obtained from Toronto Research Chemicals (Martin Ross Avenue, North York, Ontario, Canada) and LGC Standards (GmbH Mercatorstrasse, Wesel, Germany). Methanol, acetonitrile, n-hexane, and other organic solvents were purchased from Merck (Darmstadt, Germany). Formic acid and ammonium formate were purchased from Sigma-Aldrich (St. Louis, USA). Deionized water (18.2 MegaOhm.cm) was purified using a Milli-Q system (Millipore, Co., Bedford, MA, USA).

Each solid standard was accurately weighed about 10 mg and diluted by 10 mL methanol to obtain a 1000 *μ*g/mL standard solution. The stock solutions were kept in dark bottles at 4°C and to use for 1 year. The working solutions were prepared by diluting the stock solutions with methanol into the concentration of 0.01, 0.1, 0.2, 0.5, and 1 *μ*g/mL.

### 2.2. Sample Preparation

Ninety-two supplement foods and ingredients specific for the enhancement of male sexual performance were collected in local stories in Vietnam.

Samples in the form of tablets were crushed into a fine powder. In samples in the form of hard-shelled capsules or soft-gel capsules, the capsules had been removed and the inside content only has been homogenized. Each homogeneous sample was weighed 0.10 g in a 15 mL centrifuge tube by an analytical balance. Then, 4.0 mL of acetonitrile: water (1 : 1, v/v) was added and mixed well before being sonicated for 30 minutes. The extract was centrifuged for 5 minutes at a speed of 6000 rpm. The solution was separated from the residue and filtered through a polytetrafluoroethylene filter (0.2 *μ*m) before being injected into a liquid chromatography high-resolution tandem mass spectrometry (LC-HRMS). For the soft-gel capsule samples, 1.0 mL of n-hexane was added to the solution after centrifuging and mixed well to clean the oily components; only the aqueous layer was used for LC-HRMS analysis. For samples containing PDE-5i over the calibration curves, we have to reanalyze with a proper dilution factor.

### 2.3. Liquid Chromatography and High-Resolution Tandem Mass Spectrometry Condition

The sample solutions were analyzed by the UltiMate 3000 UHPLC system coupled with Q-exactive (Thermo Fisher Scientific Inc., USA). The separation was conducted on Waters BEH C18 (100 mm × 1.7 *μ*m × 2.1 mm) column with an appropriate precolumn at the temperature of 40°C. Mobile phase A was 10 mM ammonium formate and 0.1% formic acid in water; mobile phase B was 10 mM ammonium formate and 0.1% formic acid in acetonitrile: methanol (1 : 1, v/v). The gradient was 0–5 min, 2% B; 5–15 min, 2–40% B; 15–22 min, 40–95% B; 25–26 min, 95–2% B; 26–29 min, 2% B. The injection volume was 10 *μ*L. The flow rate was 0.3 mL/min.

The Q-exactive was equipped with heated electrospray ionization (HESI) source with the following parameters: HESI temperature of 320°C, the capillary temperature of 350°C, spray voltage of 5000 V, sheath gas flow of 30 arbitrary units, the auxiliary gas flow of 10 arbitrary units. The mass spectrometer was operated in the full MS/data-dependent MS/MS mode (full MS-dd-MS/MS) with the following parameters: scan range 200–2000 m/z, resolution 70,000 FWHM (defined for m/z 200; 3 Hz), automatic gain control (AGC) target 1*e*^6^, maximum inject time 20 ms, and in the dd-MS/MS mode: resolution 17,500 FWHM (defined for m/z 200; 12 Hz), AGC target 1*e*^5^, isolation window 1 m/z, normalized collision energy 40%, 70%, 100%. Full spectral information was utilized for identification and quantification. For data collection and analysis, the screening PDE-5i process was conducted by Compound discoverer 3.1 software (Thermo Fisher), and the quantitation process was conducted by TraceFinder 4.1 software (Thermo Fisher). Mass spectrometric information, including m/z of precursor and product ions of analytes, was shown in Table 1. It can be seen that the analogs of sildenafil produced the common ions at m/z 283. It is in line with previous studies that the ion is the result of cleavage of the C-S bond and loss of the ethyl group on the ethoxy substituent on the phenyl ring. For the tadalafil group, the ions at m/z 169 (pyridine-indole ring) and 135 were always recorded. The ion at m/z 344 is characterized for vardenafil and its analogs [7, 17]. Thio-sildenafil group often produces ion at m/z 299 corresponding to the cleavage of C-N bond and loss of the ethyl group on the ethoxy substituent on the phenyl ring [18].

### 2.4. Screening and Quantification of Real Samples

**Figure 1:**
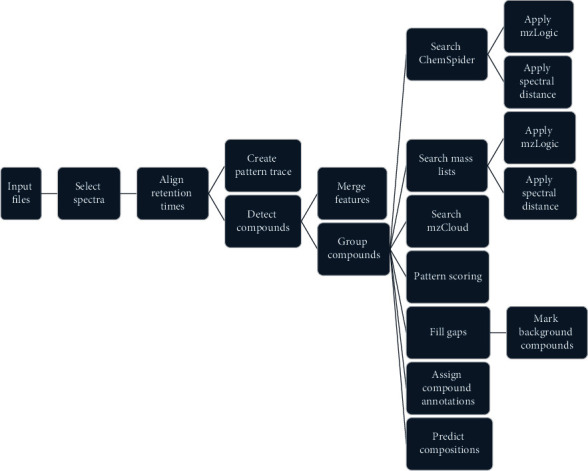
Screening workflow for unknown substances.

Real samples were first screened PDE-5i as the scheme in Figure 1. Most of the detected PDE-5i were listed in our mass spectrometry library unless the new suspected compounds were extracted and purified, and then the structure was determined by infrared spectroscopy. For the quantitative purpose, the concentration of PDE-5i in the samples was calculated by matrix match calibration curves.

## 3. Results and Discussions

### 3.1. Optimization of LC-HRMS Condition

First of all, parameters for detecting PDE-5i in Q-exactive mass spectrometer should be set up before further optimization. All PDE-5i have chemical structures suitable for being ionized by electrospray ionization source in positive mode. A 500 *μ*L mixture of 1 *μ*g/mL standard solution of PDE-5i was injected into the Q-exactive mass spectrometer to optimize ionization and detection conditions such as capillary voltage, the temperature of HESI, the temperature of ion transfer tube, S-lens level, maximum injection time, and automatic gain control. After that, the MS/MS data were recorded: full-scan mode for precursor ions and dd-MS^2^ (data-dependent MS^2^) for product ions (All ion fragmentation-AIF). The normalized collision energy (NCE) was 40, 70, 100%. The MS/MS data were compared with mzCloud Mass Spectral Library (Thermo Fisher Scientific) and the mass accuracy was less than 5 ppm, which meets the requirements of AOAC International. The MS/MS data of fifty-three standard PED-5i for identification was presented in Table 1. For the detection of PDE-5i without standard solutions, MS/MS information (Table S1) in the mzCloud Mass Spectral Library can be used.

PDE-5i are less polar compounds, so they can be analyzed by the C18 base chromatography column. Because of their similar structures, PDE-5i should be separated by a chromatography column that has a small particle size. Therefore, we chose BEH C18 (100 mm × 1.7 *μ*m × 2.1 mm, Waters, Milford, Massachusetts, USA). Commonly, mobile phases for PDE-5i analysis are acetonitrile: water and acetonitrile: methanol (1 : 1, v/v), adding additives such as formic acid, ammonium format, or both of them. We investigated and chose the mobile phase system including mobile phase A: 10 mM ammonium formate and 0.1% formic acid in water, and mobile phase B: 10 mM ammonium format and 0.1% formic acid in acetonitrile: methanol (1 : 1, v/v). The use of both ammonium formate and formic acid additives is important to gain the sensitivity of some PDE-5i and is consistent with AOAC 2015.12 method [15]. Then, the gradient was optimized and lasted 29.0 minutes to separate some isomeric PDE-5i such as Carbodenafil and Noracetildenafil, Benzamidenafil, and Tadalafil. The flow rate was 0.3 mL/min. This slow and long gradient is similar to that of the reference methods published by AOAC International and US USP [19]. The retention time of each analyte was shown in Table 1. Extracted chromatograms of PDE-5i were shown in the supplementary document (Figures S1, S2).

### 3.2. Optimization of Extraction

**Figure 2:**
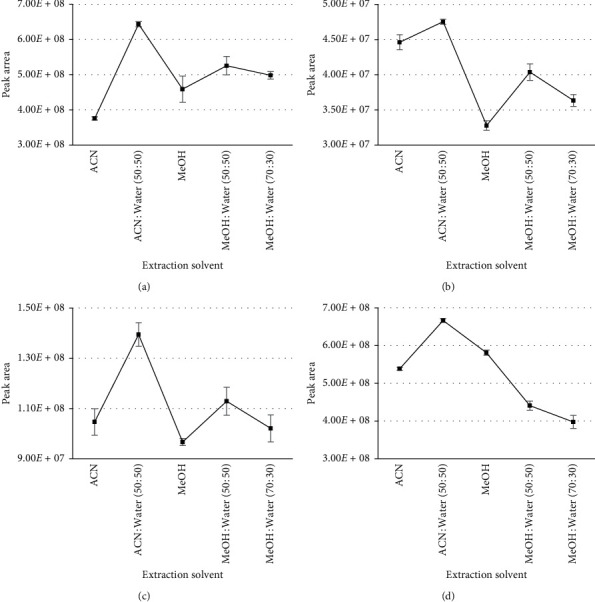
Comparison of the intensity of four representative PDE-5i with different extraction solvents. (a) Acetildenafil. (b) Acetaminotadalafil. (c) Hydroxyvardenafil. (d) Avanafil.

Referring to previous studies [7, 20], five extraction solutions were selected to examine extraction efficiency when extracting spiked samples at the concentration of 4 mg/kg in samples. The results of four representative compounds were presented in Figure 2. A one-way ANOVA test was conducted to compare the intensity of four compounds. The results (*P* value from 1.5*E*-10-4.9*E*-6 < 0.05) indicated that signal intensity changed significantly with different solvent extraction, and the mean comparison showed that the mixture of acetonitrile: water (1 : 1, v/v) gave significantly higher intensities of analytes compared to the other tested solvents. Comparing to the mixture of methanol: water (70 : 30, v:v) used by Lee et al. [17], Jeong et al. [21], or methanol used by Ren et al. [22], this method uses less organic solvent for a greener sample preparation. Thus, it was chosen to extract real samples.

**Figure 3:**
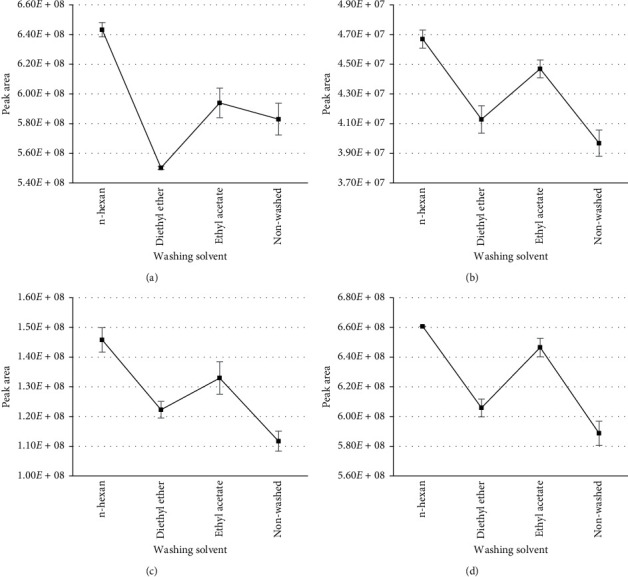
Comparison of the intensity of four representative PDE-5i after cleaning with three different solvents. (a) Acetildenafil. (b) Acetaminotadalafil. (c) Hydroxyvardenafil. (d) Avanafil.

For the soft-gel capsule sample, however, we added a second solvent to remove oily components of samples before injecting the extraction into the LC-HRMS system. Three organic solvents including n-hexane, diethyl ether, and ethyl acetate were examined, and the result was shown in Figure 3. We also compared the intensity of analytes by one-way ANOVA test. All three cleaning ways improved the intensity of analytes. The intensity of analytes in oily samples washed by n-hexane was significantly higher than that of the other solvents. Therefore, n-hexane was chosen for cleaning oily samples to reduce unwanted compounds injected into the LC-HRMS system. The use of n-hexane has not been reported before. It helps protect the ion source from fat contamination.

### 3.3. Method Validation

The developed method had been validated before applied to real samples analysis. The specificity of the method was proved by mass accuracy of precursor ions and productions and comparison between blank samples and standard materials. The validation parameters were showed in Table S2. Calibration curves of PDE-5i were constructed from 10 to 1000 ng/mL, and the regression coefficients were larger than 0.995, and relative standard deviations were less than 15%. The limit of detection and limit of quantification were 0.4 and 1.2 mg/kg, respectively. The repeatability and the recovery of the method were evaluated by analyzing spiked tablet samples and soft-gel capsule samples at three levels 30.0, 100.0, 200.0 ng/mL in solution (1.2, 4, 8 mg/kg in samples) and six repetitions. The method met the AOAC International requirement as recoveries were in the range of 80–110%, and the relative standard deviation was from 2.81 to 12.6%. The matrix effect (ME) of the method was assessed by comparing the slope of the calibration curve in solution (*A*) and one in the matrix (*A*′) as follows:

(1) $$\begin{array}{c} ME=\frac{A-A{}^{\prime }}{A}\times 100. \end{array}$$

All the compounds showed ME less than 10%. Thus, calibration curves in the standard solution can be used to calculate the concentration of PED-5i in real samples.

### 3.4. Analysis of Real Samples

For screening purposes, 92 real samples were analyzed by the developed method (*n* = 3); the screening process was conducted by Compound Discoverer 3.1 software. The spectrum was compared with the online mzCloud mass spectrometry library, Chemspider library, Mzvault library, and predicted structure. The accuracy of the process was presented by the matching index (>80%) with each library. Among 92 collected samples, twenty samples were detected containing PDE-5i; the others were not detected. In the positive samples, we identified thirteen PDE-5i already existing in the used libraries and one compound nonexisting in the used libraries. This compound was discovered as N-hydroxyethyl dithio-desethyl carbodenafil in a previous study [23]. The number of detected samples and identified PDE-5i was shown in Table 2. Ten of fourteen PDE-5i (71%) were sildenafil analogs, which is higher than the value (62%) reported by Kee et al. [4]. There were three analogs of tadalafil (21%) and only one analog of vardenafil (7.1%).

After screening, positive samples were confirmed and quantified. The concentration of PDE-5i in these samples was calculated by calibration curves and presented in Table 3. It can be seen that most of the positive samples contain one or two PDE-5i at high concentration (>1 mg/g) and some other PDE-5i at low concentration. We suppose that high concentration PDE-5i ingredients were added intentionally to the sample, and the low concentration PDE-5i may be side products in the production of the main PDE-5i ingredients. Nortadalafil, Tadalafil, and Sildenafil were often detected in real samples as the main active compounds. On the other hand, few samples (S 14 and 15) detected some PDE-5i at low concentration (much lower than the dosage using in ED treatment). The origin of PDE-5i in these samples was not clear and needed to be studied further.

## 4. Conclusion

In this study, we have developed and validated a rapid screening and quantitation method using LC-HRMS for more than 53 PDE-5i in ingredients and supplementary products for enhancing male sexual performance. The validation parameters of this method, such as LODs, LOQs, recoveries, and regression coefficients, were acceptable according to the requirement of AOAC for an analytical method. The success of this method demonstrated the utilization of the fragmentation mass spectra library for analytes confirmation. The developed method was applied to analyze 92 natural-based ingredients and supplementary products available in the Vietnam market. We had screened and detected 14 PDE-5i. The results of real samples analysis implied that the manufacturers had deceived customers by not declaring PDE-5i on the label but adding these ingredients in the products. Thus, our study provides a warning on the quality control of supplementary foods to avoid any health risks to the community.

## Figures and Tables

**Table 1:** Mass spectrometric parameters for identification and retention time of PDE-5i.

No	PDE-5i	Chemical formula	Retention time (min)	Ion type	Precursor ion (m/z)	Product ions (m/z)
1	Mirodenafil	C_26_H_37_N_5_O_5_S	7.57	M + H	532.2588	99.09167; 296.13935; 312.13427; 70.06513; 56.04948; 84.0682; 210.06619; 129.10224; 88.07569; 121.03964
2	Noracetildenafil	C_24_H_32_N_6_O_3_	13.96	M + H	453.2609	70.06513; 97.07602; 113.10732; 98.08385; 58.06513; 56.04948; 297.1346; 166.09749; 325.12952; 353.16082
3	Desmethyl fondenafil	C_23_H_30_N_6_O_3_	15.29	M + H	439.2459	339.14505; 311.11395; 396.20255; 374.42169; 319.71493; 196.38647; 165.01724; 120.37976; 99.0919; 73.11301
4	N-Octylnortadalafil	C_29_H_33_N_3_O_4_	15.64	M + H	489.3126	169.07602; 135.04406; 204.08078; 262.08626; 338.22269; 115.05423; 197.07094; 264.10191; 130.06513; 232.07569
5	Acetylvardenafil	C_25_H_34_N_6_O_3_	16.01	M + H	467.2765	169.09715; 70.06513; 84.08078; 341.16082; 111.09167; 72.08078; 127.12297; 97.07602; 112.0995; 110.06004
6	Lodenafil carbonate	C_47_H_62_N_12_O_11_S_2_	16.07	M + H	1048.642	112.0995; 82.06513; 58.06513; 97.07602; 111.09167; 56.04948; 487.2122; 83.06037; 84.08078; 283.11895
7	Hydroxyacetildenafil	C_25_H_34_N_6_O_4_	16.13	M + H	483.2714	97.07602; 70.06513; 127.08659; 143.11789; 100.07569; 297.1346; 88.07569; 166.09749; 112.0995; 128.09441
8	Carbodenafil	C_24_H_32_N_6_O_3_	16.23	M + H	453.2609	311.11387; 339.14517; 166.09749; 255.12404; 69.04472; 97.07602; 225.07709; 70.06513
9	Acetildenafil	C_25_H_34_N_6_O_3_	16.48	M + H	467.2765	111.09167; 97.07602; 70.06513; 84.08078; 72.08078; 127.12297; 112.0995; 297.1346; 56.04948; 166.09749
10	Descarbonsildenafil	C_21_H_30_N_6_O_4_S	16.59	M + H	463.2129	418.15475; 311.15069; 432.17177; 406.15494; 361.13279; 344.14795; 283.11908; 238.83536; 192.99106; 175.69979; 151.05383; 125.02768; 87.09227; 72.08158; 58.066
11	Piperiacetildenafil	C_24_H_31_N_5_O_3_	16.75	M + H	438.25	98.09643; 70.06513; 297.1346; 55.05423; 166.09749; 341.16082; 69.04472; 325.12952; 86.09643
12	Dimethylacetildenafil	C_25_H_34_N_6_O_3_	16.96	M + H	467.2765	84.08078; 127.12297; 112.0995; 111.09167; 70.06513; 297.1346; 58.06513; 166.09749; 325.1659; 410.21867
13	Hydroxyvardenafil	C_23_H_32_N_6_O_5_S	17.09	M + H	505.2228	169.09715; 344.14791; 99.09167; 110.06004; 299.11387; 123.09167; 58.06513; 56.04948; 68.01309; 82.06513
14	N-Desethylvardenafil	C_21_H_28_N_6_O_4_S	17.10	M + H	461.1966	169.09715; 344.14791; 110.06004; 299.11387; 316.11661; 123.09167; 68.01309; 82.06513; 56.04948
15	Piperazonifil	C_25_H_34_N_6_O_4_	17.10	M + H	483.2726	465.26167; 436.22269; 429.52412; 408.22737; 380.20885; 339.1819; 297.13488; 266.45598; 244.12189; 203.11833; 153.1027; 127.08668; 99.0923; 72.08144
16	Vardenafil	C_23_H_32_N_6_O_4_S	17.20	M + H	489.2279	169.09715; 344.14791; 110.06004; 299.11387; 72.08078; 123.09167; 70.06513; 376.1074; 68.01309; 113.10732
17	Avanafil	C_23_H_26_ClN_7_O_3_	17.33	M + H	484.1858	155.02582; 375.12184; 105.03349; 77.03858; 95.04914; 53.03858; 357.11128; 233.1033; 67.05423; 221.1033
18	Isosildenafil	C_22_H_30_N_6_O_4_S	17.35	M + H	475.2122	58.06513; 99.09167; 283.11895; 100.0995; 56.04948; 253.072; 70.06513; 311.15025; 225.07709
19	Hydroxyhomosildenafil	C_23_H_32_N_6_O_5_S	17.36	M + H	505.2228	99.09167; 70.06513; 58.06513; 84.0682; 97.07602; 283.11895; 88.07569; 129.10224; 112.0995; 311.15025
20	N-Desmethylsildenafil	C_21_H_28_N_6_O_4_S	17.37	M + H	461.1966	85.07602; 283.11895; 311.15025; 56.04948; 299.10868; 225.07709; 254.07983; 253.072; 377.1278; 344.14791
21	Sildenafil	C_22_H_30_N_6_O_4_S	17.39	M + H	475.2122	58.06513; 100.0995; 99.09167; 56.04948; 283.11895; 70.06513; 311.15025; 225.07709; 299.11387
22	Homosildenafil	C_23_H_32_N_6_O_4_S	17.47	M + H	489.2279	72.08078; 58.06513; 99.09167; 113.10732; 70.06513; 283.11895; 84.08078; 71.07295; 114.11515; 311.15025
23	Acetaminotadalafil	C_23_H_20_N_4_O_5_	17.60	M + H	433.1507	204.08078; 262.08626; 135.04406; 205.0886; 233.08352; 232.07569; 169.07602; 191.07295; 263.09408; 250.08626
24	Aminotadalafil	C_21_H_18_N_4_O_4_	17.60	M + H	391.1401	204.08078; 135.04406; 262.08626; 233.08352; 169.07602; 232.07569; 250.08626; 191.07295; 203.07295
25	Sildenafil N-oxide	C_22_H_30_N_6_O_5_S	17.60	M + H	491.2071	99.09167; 56.04948; 70.06513; 404.1387; 344.14791; 58.06513; 97.07602; 283.11895; 311.15025; 377.1278
26	Cyclopentylnafil	C_26_H_36_N_6_O_4_S	17.70	M + H	529.2592	461.19682; 377.13029; 344.1461; 313.16608; 277.28223; 237.59493; 210.18739; 169.09731; 142.733; 98.09704; 75.59057
27	Dimethylsildenafil	C_23_H_32_N_6_O_4_S	17.71	M + H	489.2279	99.09167; 71.07295; 56.04948; 113.10732; 70.06513; 283.11895; 311.15025; 84.08078; 377.1278; 225.07709
28	Nortadalafil	C_21_H_17_N_3_O_4_	17.77	M + H	376.1292	204.08078; 262.08626; 135.04406; 233.08352; 232.07569; 169.07602; 191.07295; 254.0924; 250.08626
29	Udenafil	C_25_H_36_N_6_O_4_S	17.98	M + H	517.2592	84.08078; 112.11208; 283.11895; 58.06513; 325.1659; 299.11387; 81.06988; 255.12404; 79.05423; 82.06513
30	Benzamidenafil	C_19_H_23_N_3_O_6_	18.02	M + H	390.166	151.07536; 107.04914; 135.04406; 91.05423; 79.05423; 105.03349; 90.0464; 136.05188; 65.03858; 93.03349
31	Norneovardenafil	C_18_H_20_N_4_O_4_	18.07	M + H	357.1557	169.07602; 110.06004; 329.12443; 328.11661; 123.09167; 68.01309; 300.08531; 55.05423; 82.06513; 95.06037
32	Propoxyphenyl-homohydroxysildenafil	C_24_H_34_N_6_O_5_S	18.13	M + H	519.2384	99.09167; 70.06513; 283.11895; 84.0682; 97.07602; 299.11387; 129.10224; 88.07569; 112.0995; 255.12404
33	O-desethyl-o-propyl sildenafil	C_23_H_32_N_6_O_4_S	18.14	M + H	489.2285	447.1196; 416.97849; 391.14451; 347.08125; 325.16612; 283.11909; 252.20022; 230.78935; 193.95794; 163.05384; 107.28071; 100.10004; 91.75806; 70.06595; 58.06599
34	2-Hydroxypropyl nortadalafil	C_24_H_23_N_3_O_5_	18.20	M + H	434.1711	135.04406; 169.07602; 204.08078; 262.08626; 284.13935; 197.07094; 130.06513; 115.05423; 232.07569; 312.13427
35	Propoxyphenyl aildenafil	C_24_H_34_N_6_O_4_S	18.39	M + H	503.2447	461.19514; 391.14362; 347.08197; 325.16609; 283.11925; 256.09442; 189.66799; 159.62481; 137.61562; 113.1077; 99.09223; 91.76256; 71.07376
36	Acetil acid	C_18_H_20_N_4_O_4_	18.45	M + H	357.1557	285.1345; 300.08487; 313.16528; 273.23201; 234.78161; 329.12441; 57.55269; 76.77712; 91.7671; 128.3394; 166.09776
37	Tadalafil	C_22_H_19_N_3_O_4_	18.69	M + H	390.1448	204.08078; 135.04406; 262.08626; 169.07602; 205.0886; 232.07569; 233.08352; 240.11314; 268.10805; 250.08626
38	Depiperazino-thiosildenafil	C_17_H_20_N_4_O_4_S_2_	18.80	M + H	409.1012	381.06896; 365.03707; 352.03045; 328.13556; 300.10448; 272.07283; 253.43238; 218.38203; 200.90222; 182.07341; 146.98571; 130.30821; 91.75983; 69.50264
39	Mutaprodenafil	C_27_H_35_N_9_O_5_S_2_	18.95	M + H	630.2282	142.00711; 602.23247; 560.22363; 516.1504; 489.22772; 439.15549; 404.13937; 377.12856; 344.14797; 312.15851; 288.21153; 219.20777; 163.22714; 113.10774; 84.98616
40	Gendenafil	C_19_H_22_N_4_O_3_	19.24	M + H	355.1765	327.14517; 285.1346; 298.10604; 256.09548; 311.11387; 69.04472; 120.04439; 154.0611; 313.1659; 166.09749
41	Hydroxychlorodenafil	C_19_H_23_ClN_4_O_3_	19.26	M + H	391.1531	313.12952; 285.1346; 363.12184; 256.09548; 120.04439; 69.04472; 166.09883; 78.99452; 327.14517; 255.08765
42	Hydroxythiovardenafil	C_23_H_32_N_6_O_4_S_2_	19.57	M + H	521.1999	167.06375; 360.12506; 99.09167; 315.09037; 138.02462; 150.10257; 58.06513; 299.09611; 70.06513; 332.09307
43	Chloropretadalafil	C_22_H_19_ClN_2_O_5_	19.65	M + H	427.1055	135.04406; 274.08559; 204.08078; 216.08078; 189.06988; 262.08674; 244.0735; 302.08117
44	Chlorodenafil	C_19_H_21_ClN_4_O_3_	19.79	M + H	389.1375	361.10619; 285.1346; 311.11387; 154.0611; 166.09749; 69.0573; 256.09548; 76.97887; 165.0183
45	Benzylsildenafil	C_28_H_34_N_6_O_4_S	20.01	M + H	551.2435	91.05423; 65.03858; 134.09643; 377.1278
46	Nitrodenafil	C_17_H_19_N_5_O_4_	20.09	M + H	358.151	330.11968; 316.11661; 154.0611; 256.09548; 68.0369; 255.08765; 313.11694; 227.09274; 269.1033; 136.05054
47	Pseudovardenafil	C_22_H_29_N_5_O_4_S	20.21	M + H	460.2013	169.09715; 110.06004; 344.14791; 299.11387; 123.09167; 284.12678; 68.01309; 82.06513; 55.05423; 95.06037
48	Imidazosagatriazinone	C_17_H_20_N_4_O_2_	20.32	M + H	313.1659	285.1346; 256.09548; 120.04439; 68.0369; 255.08765; 241.072; 269.1033; 69.04472; 154.0611; 94.02874
49	Propoxyphenylthio-hydroxyhomosildenafil	C_24_H_34_N_6_O_4_S_2_	20.55	M + H	535.2156	99.09167; 70.06513; 56.04948; 299.09611; 58.06513; 84.0682; 129.10224; 315.09037; 88.07569; 271.10119
50	Thiohomosildenafil	C_23_H_32_N_6_O_3_S_2_	20.55	M + H	505.205	72.08078; 99.09167; 113.10732; 56.04948; 299.09611; 70.06513; 84.08078; 327.12741; 71.07295; 355.15806
51	Hydroxythio-homosildenafil	C_23_H_32_N_6_O_4_S_2_	20.56	M + H	521.1999	99.09167; 70.06513; 58.06513; 84.0682; 299.09611; 129.10224; 97.07602; 88.07569; 327.12741; 112.0995
52	Norneosildenafil	C_22_H_29_N_5_O_4_S	20.64	M + H	460.2013	283.11895; 84.08078; 299.09611; 311.15025; 154.0611; 316.11661; 255.12404; 344.14791; 166.09749
53	Thiosildenafil	C_23_H_32_N_6_O_3_S_2_	21.47	M + H	505.205	99.09167; 71.07295; 299.09611; 113.10732; 56.04948; 70.06513; 327.12741; 84.08078; 241.0542; 298.08828

**Table 2:** Result of screening PDE-5i in 92 samples.

PDE-5i	Number of detected samples
Nortadalafil	11
Tadalafil	10
Sildenafil	9
Hydroxyhomosildenafil	6
Hydroxythiohomosildenafil	6
Homosildenafil	6
Thiohomosildenafil	1
Sildenafil N-oxyde	2
Chloropredadalafil	1
Propoxyphenyl-homohydroxysildenafil	1
Propoxyphenylaildenafil	1
Hydroxythiovardenafil	1
Methisosildenafil	1
N-hydroxyethyl dithio-desethyl carbodenafil	2

**Table 3:** Concentration of PDE-5i detected in real samples.

Sample	PDE-5i	Concentration (mg/g)
S 1	Nortadalafil	50.0 ± 0.13
	Tadalafil	11.6 ± 0.05
	Sildenafil-N-oxide	∼0.073
S 2	Nortadalafil	4.41 ± 0.05
	Chloropretadalafil	∼0.006
S 3	Sildenafil	22.2 ± 0.11
	Tadalafil	0.39 ± 0.007
	Sildenafil-N-oxide	∼0.06
S 4	Propoxyphenylaildenafil	1.02 ± 0.06
	Thiohomosildenafil	0.78 ± 0.005
	Homosildenafil	∼0.031
	Methisosildenafil	∼0.03
S 5–9	Hydroxyhomosildenafil	1.05–20.1
	Hydroxythiohomosildenafil	0.78–22.5
S 10	Tadalafil	∼0.08
S 11	Tadalafil	10.2 ± 0.05
	Chloropretadalafil	5.25 ± 0.06
S 12	Nortadalafil	12.2 ± 0.05
	Chloropretadalafil	0.25 ± 0.06
S 13	Nortadalafil	10.2 ± 0.04
	Chloropretadalafil	5.25 ± 0.10
S 14	Acetil acid	0.13 ± 0.06
S 15	Tadalafil	0.12 ± 0.05
S 16	Sildenafil	5.25 ± 0.11
	Tadalafil	4.77 ± 0.15
	Aminotadalafil	0.52 ± 0.05
	Sildenafil N-oxyde	0.56 ± 0.06
S 17	Nortadalafil	0.52 ± 0.06
	Chloropretadalafil	4.77 ± 0.10
S 18	Nortadalafil	12.1 ± 0.06
M 1	Nortadalafil	170.0 ± 1.22
M 2	Hydroxythiohomosildenafil	226.8 ± 1.36
	Hydroxythiovardenafil	216.8 ± 2.00
	Propoxyphenyl-homohydroxysildenafil	0.13 ± 0.06
	Hydroxyhomo-sildenafil	∼0.048
	Hydroxyvardenafil	∼0.047

S: supplementary food, M: medicine ingredient, “S 5–9” indicates samples: S 5, S 6, S 7, S 8, S 9.

## Data Availability

The data used to support the findings of this study are available within the article, the support information in word form, and from the corresponding author upon request.
