# Supplementary material for: Rapid Screening and Quantitative Determination of Illegal Phosphodiesterase Type 5 Inhibitors (PDE-5i) in Herbal Dietary Supplements
Source: J Anal Methods Chem. 2021 May 5;2021:5579500. doi: 10.1155/2021/5579500 (PMC8116155; doi:10.1155/2021/5579500)
Supplement: Supplementary Materials — Supplementary information is provided in word form. [file 5579500.f1.docx]

Supplementary Information

**Journal of Analytical Methods in Chemistry**

**Screening and quantitative determination of phosphodiesterase type 5 inhibitors (PDE-5i) in herbal dietary supplements**

Thi Oanh Nguyen,^1,2^ Cao-Son Tran,^3^ Thi Thu Hang Do,^3^ Nguyen Thi Minh Hoa,^3^ Quang-Dong Bui,^3^ Cao-Tien Bui,^3^ Hong-Ngoc Nguyen,^3^ Thu-Hien Dang,^3^ Thi Anh Huong Nguyen,^2^ Thi Hong Hao Le^2,3^

^1^Vietnam Food Administration, Hanoi 10000, Viet Nam.
^2^University of Science, Vietnam National University, Hanoi 10000, Viet Nam.
^3^National Institute for Food Control, Hanoi 10000, Viet Nam.

Co-correspondence should be addressed to Thi Hong Hao Le ([lethihonghao@yahoo.com](mailto:lethihonghao@yahoo.com)) and Cao-Son Tran ([sontc@nifc.gov.vn](mailto:sontc@nifc.gov.vn))

**Table of content**

[Table S1: Mass spectrometric information including m/z of precursor and product ions of PDE-5i without standard materials 1](#_Toc68688165)

[Table S2: Linearity, precision, LOD, LOQ, average recovery, and matrix effect of PDE-5i 3](#_Toc68688166)

[Figure S1: Extracted chromatogram of some PDE-5i at a concentration of 30ng/mL 7](#_Toc68688173)

[Figure S2: Extracted chromatogram of some PDE-5i at a concentration of 30 ng/mL (cont) 8](#_Toc68688174)

Table S1: Mass spectrometric information including m/z of precursor and product ions of PDE-5i without standard materials

| No | PDE-5i | Chemical formula | Ion type | Precursor ion (m/z) | Product ion (m/z) |
| --- | --- | --- | --- | --- | --- |
|  | Desmethylthiosildenafil | C_21_H_28_N_6_O_3_S_2_ | M+H | 477,17371 | 85,07602; 299,0961; 327,1274; 56,04948; 269,0485; 241,0542; 298,0883; 271,1005; 227,0379 |
|  | Desmethylenetadalafil | C_21_H_19_N_3_O_4_ | M+H | 378,14483 | 204,0808; 204,0808; 123,0441; 250,0863; 180,0808; 169,076; 234,0913; 130,0651; 256,1081; 238,0863 |
|  | Dinitrodenafil | C_17_H_18_N_6_O_6_ | M+H | 403,13606 | 283,119; 358,102; 375,1048; 225,0771; 361,1017; 154,0611; 344,099; 344,099; 217,1084; 253,072 |
|  | Gisadenafil | C_23_H_33_N_7_O_5_S | M+H | 520,23366 | 72,08078; 111,0917; 58,06513; 99,09167; 56,04948; 83,06037; 70,06513; 113,1073; 332,1353 |
|  | N-Butyltadalafil | C_25_H_25_N_3_O_4_ | M+H | 432,19178 | 135,0441; 204,0808; 169,076; 262,0863; 205,0886; 250,0863; 282,1601; 232,0757; 191,073; 264,1019 |
|  | N-Ethyltadalafil | C_23_H_21_N_3_O_4_ | M+H | 404,16048 | 135,0441; 204,0808; 169,076; 262,0863; 205,0886; 232,0757; 250,0863; 233,0835; 254,1288; 191,073 |
|  | Propoxyphenyl-thioaildenafil | C_24_H_34_N_6_O_3_S_2_ | M+H | 519,22066 | 477,1745; 458,1180; 407,1211; 341,1433; 315,0915; 299,0964; 271,2639; 245,5092; 195,9292; 149,2379; 135,6055 |
|  | Propoxyphenyl-sildenafil | C_23_H_32_N_6_O_4_S | M+H | 489,22785 | 58,06513; 283,119; 100,0995; 99,09167; 299,1172; 56,04948; 255,124; 225,077; 166,0975; 97,07602 |
|  | Propoxyphenyl-thiosildenafil | C_23_H_32_N_6_O_3_S_2_ | M+H | 505,20501 | 58,06513; 299,0961; 99,09167; 100,0995; 313,1118; 56,04948; 70,06513; 315,0917; 329,1067; 271,1012 |
|  | Pyrazole N-demethylsildenafil | C_21_H_28_N_6_O_4_S | M+H | 461,19655 | 297,1346; 313,1295; 58,06513; 101,1073; 241,1084; 269,1033; 99,09167; 285,0982; 70,06513; 212,0700 |
|  | Thiosildenafil | C_22_H_30_N_6_O_3_S_2_ | M+H | 491,18936 | 58,06513; 99,09167; 100,0995; 299,0961; 56,04948; 70,06513; 341,1424; 327,1274; 85,07602; 312,1033 |
|  | Vardenafil N-oxide | C_23_H_32_N_6_O_5_S | M+H | 505,22277 | 169,0972; 344,1479; 477,1915; 70,06513; 113,1073; 110,06; 377,1278; 123,0917; 98,08385; 56,04948 |
|  | Vardenafil oxopiperazine | C_21_H_26_N_6_O_5_S | M+H | 475,17582 | 169,0972; 344,1479; 110,06; 299,1139; 123,0917; 316,1166; 68,01309; 82,06513; 55,05423; 95,06037 |
|  | Zaprinast | C_13_H_13_N_5_O_2_ | M+H | 272,11420 | 120,0444; 202,0611; 230,0673; 92,04948; 157,0396; 104,0495; 55,02907; 65,03858; 159,0553; 132,0444 |

Table S2: Linearity, precision, LOD, LOQ, average recovery, and matrix effect of PDE-5i

| **No** | **PDE-5i** | **Calibration curve** | **Precision (RSD%)** | **LOD (mg/kg)** | **LOQ (mg/kg)** | **Average recovery (%)** | **Matrix effect (ME %)** |
| --- | --- | --- | --- | --- | --- | --- | --- |
| **1** | Mirodenafil | y=6.178*e^6 X - 5.349*e^6 (R^2=0.9998) | 2.46 | 0.4 | 1.32 | 102.31 | 6.67 |
| **2** | Noracetildenafil | y=1.654*e^7 X - 1.578*e^8 (R^2=0.9982) | 3.22 | 0.4 | 1.32 | 102.73 | 5.12 |
| **3** | Desmethyl fondenafil | y=1.049*e^7 X - 6.371*e^8 (R^2=0.9986) | 2.52 | 0.4 | 1.32 | 104.62 | 4.58 |
| **4** | N-Octylnortadalafil | y=1.335*e^6 X - 8.052*e^6 (R^2=0.9998) | 1.12 | 0.4 | 1.32 | 106.18 | 3.15 |
| **5** | Acetylvardenafil | y=2.136*e^6 X - 7.494*e^6 (R^2=0.9998) | 1.68 | 0.4 | 1.32 | 105.05 | 5.57 |
| **6** | Lodenafil carbonate | y=8.638*e^6 X - 9.313*e^6 (R^2=0.9996) | 1.30 | 0.4 | 1.32 | 101.81 | 5.08 |
| **7** | Hydroxyacetildenafil | y=2.289*e^6 X - 1.166*e^6 (R^2=0.9996) | 1.73 | 0.4 | 1.32 | 101.74 | 6.89 |
| **8** | Carbodenafil | y=5.815*e^6 X - 7.817*e^6 (R^2=0.9978) | 2.33 | 0.4 | 1.32 | 103.36 | 4.70 |
| **9** | Acetildenafil | y=6.302*e^6 X - 2.166*e^7 (R^2=0.9995) | 2.76 | 0.4 | 1.32 | 102.77 | 5.59 |
| **10** | Descarbonsildenafil | y=2.345*e^6 X - 1.664*e^7 (R^2=0.9975) | 1.34 | 0.4 | 1.32 | 103.50 | 7.24 |
| **11** | Piperiacetildenafil | y=1.262*e^6 X - 8.601*e^6 (R^2=1.0000) | 1.45 | 0.4 | 1.32 | 99.91 | 4.51 |
| **12** | Dimethylacetildenafil | y=4.121*e^6 X - 2.660*e^7 (R^2=0.9998) | 4.51 | 0.4 | 1.32 | 102.28 | 4.00 |
| **13** | Hydroxyvardenafil | y=2.709*e^6 X - 8.784*e^6 (R^2=0.9986) | 2.21 | 0.4 | 1.32 | 100.58 | 5.76 |
| **14** | N-Desethylvardenafil | y=5.103*e^5 X - 4.694*e^6 (R^2=0.9991) | 1.47 | 0.4 | 1.32 | 97.64 | 5.92 |
| **15** | Piperazonifil | y=5.670*e^4 X - 8.710*e^4 (R^2=0.9983) | 1.45 | 0.4 | 1.32 | 98.16 | 7.12 |
| **16** | Vardenafil | y=1.064*e^5 X - 1.202*e^6 (R^2=1.0000) | 3.59 | 0.4 | 1.32 | 100.02 | 6.46 |
| **17** | Avanafil | y=3.709*e^4 X - 4.588*e^5 (R^2=0.9999) | 1.72 | 0.4 | 1.32 | 100.47 | 6.67 |
| **18** | Isosildenafil | y=6.451*e^6 X + 1.886*e^7 (R^2=0.9981) | 2.55 | 0.4 | 1.32 | 102.34 | 5.05 |
| **19** | Hydroxyhomosildenafil | y=1.076*e^6 X - 4.591*e^6 (R^2=0.9992) | 2.27 | 0.4 | 1.32 | 98.51 | 4.94 |
| **20** | N-Desmethylsildenafil | y=3.551*e^5 X - 3.350*e^6 (R^2=0.9976) | 4.48 | 0.4 | 1.32 | 101.59 | 4.02 |
| **21** | Sildenafil | y=9.580*e^4 X - 1.023*e^6 (R^2=0.9964) | 2.73 | 0.4 | 1.32 | 102.48 | 4.30 |
| **22** | Homosildenafil | y=1.154*e^6 X - 1.008*e^7 (R^2=0.9976) | 1.67 | 0.4 | 1.32 | 102.53 | 4.21 |
| **23** | Acetaminotadalafil | y=1.838*e^6 X - 1.181*e^7 (R^2=0.9995) | 1.82 | 0.4 | 1.32 | 102.07 | 5.88 |
| **24** | Aminotadalafil | y=2.201*e^6 X - 2.879*e^7 (R^2=1.0000) | 2.77 | 0.4 | 1.32 | 98.55 | 3.15 |
| **25** | Sildenafil N-oxide | y=6.314*e^6 X - 6.025*e^7 (R^2=0.9996) | 2.05 | 0.4 | 1.32 | 98.03 | 3.48 |
| **26** | Cyclopentylnafil | y=2.003*e^6 X - 1.611*e^7 (R^2=0.9997) | 1.97 | 0.4 | 1.32 | 101.99 | 5.90 |
| **27** | Dimethylsildenafil | y=1.930*e^6 X - 7.092*e^6 (R^2=0.9940) | 2.84 | 0.4 | 1.32 | 102.31 | 4.56 |
| **28** | Nortadalafil | y=4.368*e^4 X - 6.043*e^5 (R^2=0.9993) | 2.18 | 0.4 | 1.32 | 104.51 | 5.78 |
| **29** | Udenafil | y=1.068*e^7 X - 3.943*e^7 (R^2=0.9949) | 1.95 | 0.4 | 1.32 | 104.87 | 7.57 |
| **30** | Benzamidenafil | y=1.068*e^7 X - 3.943*e^7 (R^2=0.9949) | 1.34 | 0.4 | 1.32 | 98.94 | 5.92 |
| **31** | Norneovardenafil | y=2.566*e^5 X - 3.393*e^6 (R^2=0.9991) | 2.37 | 0.4 | 1.32 | 106.24 | 2.63 |
| **32** | Propoxyphenyl-homohydroxysildenafil | y=5.827*e^5 X - 5.275*e^6 (R^2=1.0000) | 2.16 | 0.4 | 1.32 | 108.63 | 3.95 |
| **33** | O-desethyl-o-propyl sildenafil | y=4.917*e^6 X - 8.105*e^7 (R^2=0.9913) | 2.93 | 0.4 | 1.32 | 106.67 | 3.06 |
| **34** | 2-Hydroxypropyl nortadalafil | y=1.702*e^6 X - 1.977*e^7 (R^2=0.9998) | 2.00 | 0.4 | 1.32 | 107.25 | 4.07 |
| **35** | Propoxyphenyl aildenafil | y=1.475*e^6 X - 1.663*e^7 (R^2=0.9999) | 2.38 | 0.4 | 1.32 | 104.76 | 6.49 |
| **36** | Acetil acid | y=8.306*e^6 X - 1.602*e^7 (R^2=0.9989) | 3.08 | 0.4 | 1.32 | 108.38 | 5.48 |
| **37** | Tadalafil | y=3.561*e^6 X - 3.475*e^7 (R^2=1.0000) | 1.92 | 0.4 | 1.32 | 102.85 | 3.15 |
| **38** | Depiperazino-thiosildenafil | y=2.259*e^5 X - 2.981*e^6 (R^2=0.9987) | 1.70 | 0.4 | 1.32 | 100.18 | 3.41 |
| **39** | Mutaprodenafil | y=2.012*e^5 X - 1.398*e^6 (R^2=0.9997) | 2.45 | 0.4 | 1.32 | 99.10 | 6.09 |
| **40** | Gendenafil | y=2.515*e^6 X - 1.670*e^7 (R^2=0.9975) | 2.52 | 0.4 | 1.32 | 100.06 | 5.19 |
| **41** | Hydroxychlorodenafil | y=1.343*e^6 X - 8.663*e^6 (R^2=1.0000) | 3.03 | 0.4 | 1.32 | 99.67 | 3.45 |
| **42** | Hydroxythiovardenafil | y=4.211*e^6 X - 2.580*e^7 (R^2=0.9998) | 2.75 | 0.4 | 1.32 | 101.99 | 3.41 |
| **43** | Chloropretadalafil | y=2.755*e^6 X - 8.776*e^6 (R^2=0.9986) | 2.84 | 0.4 | 1.32 | 92.02 | 4.85 |
| **44** | Chlorodenafil | y=5.535*e^5 X - 4.205*e^6 (R^2=0.9991) | 2.15 | 0.4 | 1.32 | 94.91 | 4.04 |
| **45** | Benzylsildenafil | y=5.220*e^4 X - 8.161*e^4 (R^2=0.9983) | 2.10 | 0.4 | 1.32 | 95.79 | 5.99 |
| **46** | Nitrodenafil | y=1.052*e^5 X - 1.212*e^6 (R^2=1.0000) | 1.71 | 0.4 | 1.32 | 97.36 | 5.33 |
| **47** | Pseudovardenafil | y=2.030*e^6 X - 6.955*e^6 (R^2=0.9940) | 1.83 | 0.4 | 1.32 | 94.92 | 6.05 |
| **48** | Imidazosagatriazinone | y=4.555*e^4 X - 6.122*e^5 (R^2=0.9993) | 1.79 | 0.4 | 1.32 | 92.43 | 6.42 |
| **49** | Propoxyphenylthio-hydroxyhomosildenafil | y=1.058*e^7 X - 4.043*e^7 (R^2=0.9949) | 2.50 | 0.4 | 1.32 | 97.89 | 3.92 |
| **50** | Thiohomosildenafil | y=1.078*e^7 X - 3.953*e^7 (R^2=0.9949) | 1.34 | 0.4 | 1.32 | 100.23 | 4.28 |
| **51** | Hydroxythio-homosildenafil | y=2.766*e^5 X - 3.287*e^6 (R^2=0.9991) | 1.48 | 0.4 | 1.32 | 102.36 | 6.51 |
| **52** | Norneosildenafil | y=6.027*e^5 X - 5.185*e^6 (R^2=1.0000) | 2.51 | 0.4 | 1.32 | 102.87 | 4.82 |
| **53** | Thiosildenafil | y=4.922*e^6 X - 8.088*e^7 (R^2=0.9913) | 1.94 | 0.4 | 1.32 | 102.96 | 4.63 |


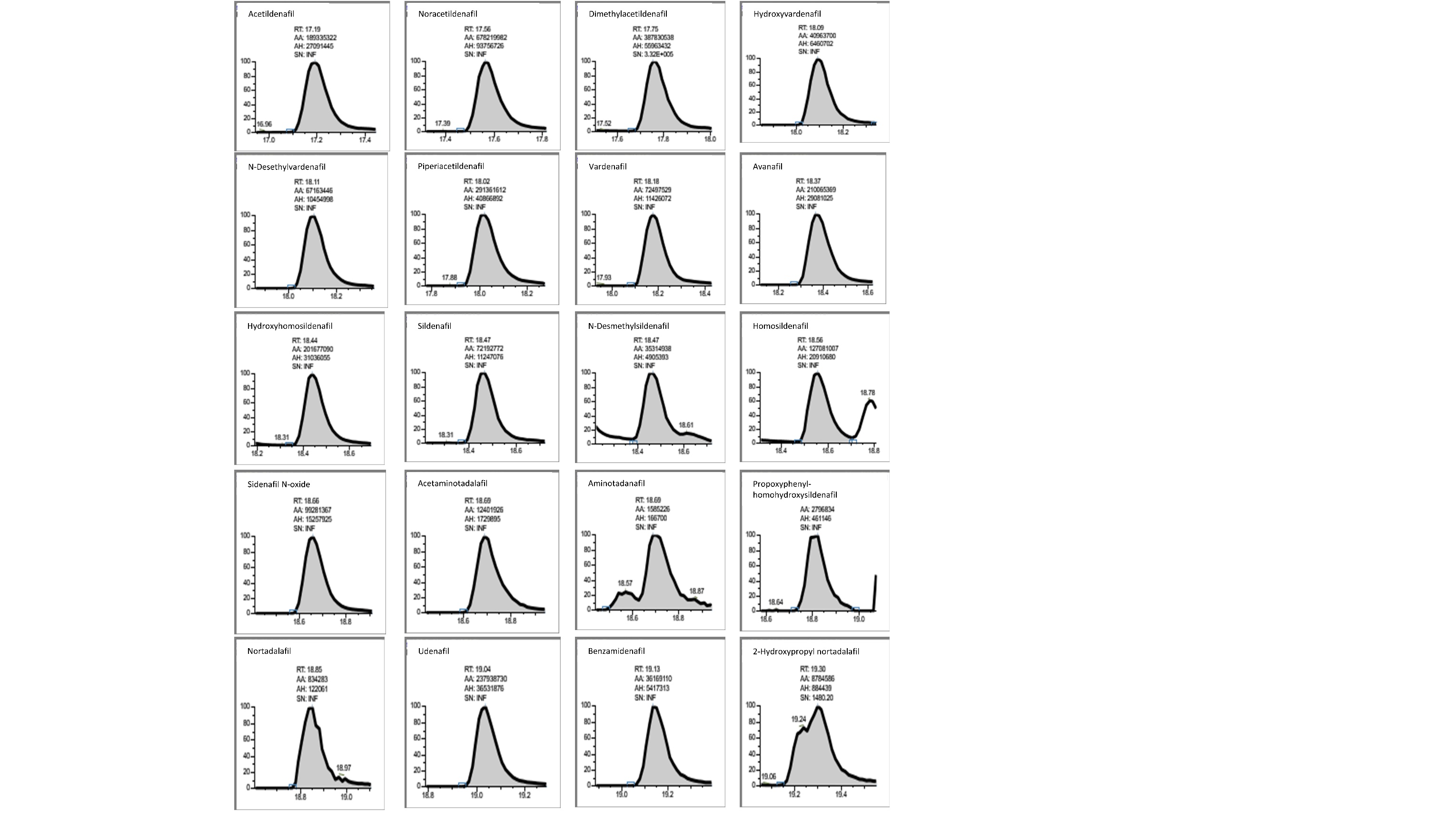


Figure S1: Extracted chromatogram of some PDE-5i at a concentration of 30ng/mL


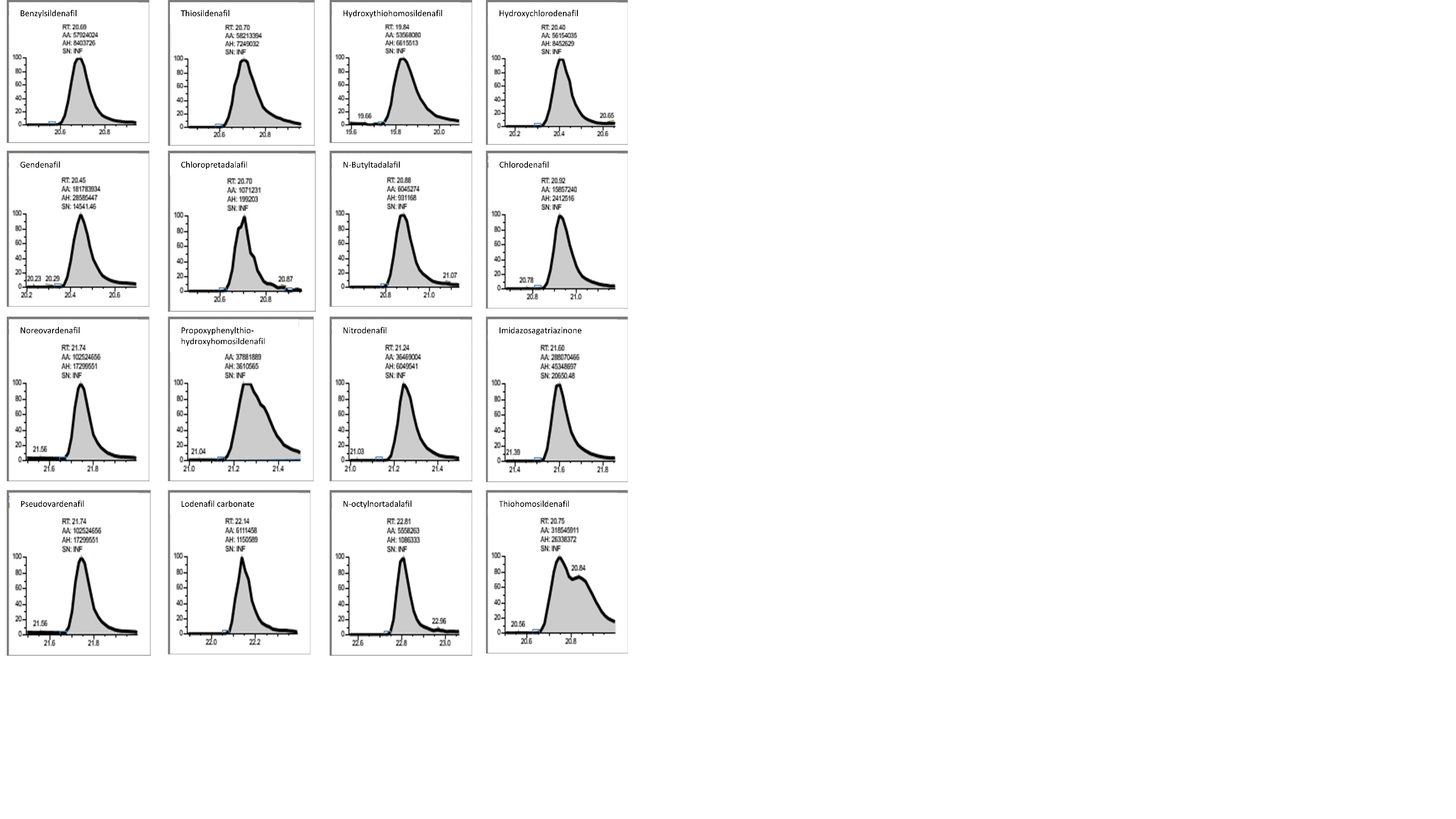


Figure S2: Extracted chromatogram of some PDE-5i at a concentration of 30 ng/mL (cont)
